# Seroprevalence of SARS-CoV-2 antibodies, associated factors, experiences and attitudes of nursing home and home healthcare employees in Switzerland

**DOI:** 10.1186/s12879-022-07222-8

**Published:** 2022-03-16

**Authors:** Erin A. West, Olivia J. Kotoun, Larissa J. Schori, Julia Kopp, Marco Kaufmann, Manuela Rasi, Jan Fehr, Milo A. Puhan, Anja Frei, the Corona Immunitas Research Group

**Affiliations:** Epidemiology, Biostatistics and Prevention Institute, University of Zurich, Zurich, Switzerland

**Keywords:** SARS-CoV-2, Seroprevalence, Outbreak, Nursing homes, Healthcare employees, At home care, Behavioural, Pandemic

## Abstract

**Background:**

Many studies in hospital settings exist and have shown healthcare employees to be particularly exposed to SARS-CoV-2. While research focused on hospital staff, little evidence exists for employees in nursing homes and home care. The aims of this study were to assess the seroprevalence in nursing homes and home care employees in the Canton of Zurich, compare it to the general population, assess factors associated with seropositivity and explore the perspective of the employees regarding how the pandemic changed their daily work.

**Methods:**

This cross-sectional study is part of the national Corona Immunitas research program of coordinated, seroprevalence studies in Switzerland. Six nursing homes and six home healthcare organizations providing at home care services in Zurich were selected and 296 and 131 employees were recruited, respectively. Assessments included standardized questionnaires, blood sampling for antibodies, and additional work-specific questions. All participants were recruited between 21st September and 23rd October 2020, before the second wave of the pandemic hit Switzerland, and were possibly exposed to SARS-CoV-2 at their work during the first wave in spring 2020.

**Results:**

Seroprevalence of SARS-CoV-2 was 14.9% (95% CI 11.1%-19.6%; range 3.8% to 24.4%) for nursing home employees and 3.8% (95% CI 1.4–9.1%; range 0% to 10%) for home healthcare employees, compared to the general population of Zurich at 3.5% in September 2020 for those aged 20–64. Nurses were 2.6 times more likely to have SARS-CoV-2 antibodies than those employees who were not nurses (95% CI 1.1–6.2). The employees (nursing homes vs. home healthcare) perceived the implementation of general safety measures (44.9% vs. 57.3%) and wearing masks during work (36.8% vs. 43.5%), especially due to the limited communication with residents/clients, as the most crucial changes.

**Conclusions:**

Nursing home employees who worked through SARS-CoV-2 outbreaks at their work were substantially more affected by SARS-CoV-2 infection compared to the general population and to home healthcare employees who similarly worked through outbreaks in their communities. Employees reported that important resources to cope with the burdensome changes they perceived in their daily work were personal resources and team support.

***Trial registration*:**

Current Controlled Trials ISRCTN18181860 dated 09/07/2020. Retrospectively registered.

**Supplementary Information:**

The online version contains supplementary material available at 10.1186/s12879-022-07222-8.

## Background

The illness coronavirus disease 2019 (COVID-19) caused by severe acute respiratory syndrome coronavirus 2 infection (SARS-CoV-2) was first reported in Switzerland in February 2020 [1]. By December 2021, there were over 1,200,000 confirmed cases and over 12,000 deaths. Based on the current, ongoing seroprevalence studies of Corona Immunitas as well as research done in other countries, it is estimated that the true number of cases in Switzerland would be over three million—three times the number of those reported cases [2, 3]. Much of the current literature, particularly the early studies at the onset of the pandemic, focus on healthcare employees in the hospital setting, but not as much is known about the potential increased risk of exposure to those healthcare employees in the nursing home and home healthcare setting [4–6]. Home healthcare and nursing home employees are at particularly high risk to SARS-CoV-2 infection due to their close contact with potentially infected patients, residents or clients [4, 7, 8]. They further suffer a burden of increased preventative measures in their daily working life in order to help protect both themselves and their clients/patients against SARS-CoV-2 infection [9] as well as increased stress over working the ‘front lines’ during this global pandemic [10].

Due to the potential for asymptomatic carriers of SARS-CoV-2 [11–13], it is challenging to fully understand how much more this population is at risk of exposure. Ascertaining the prevalence for this vulnerable population is helpful for better understanding their risk. A living systematic review of the prevalence of healthcare employees with confirmed SARS-CoV-2 infection in Europe, the United States and Asia found that prevalence can vary considerably, from 0.4% in Spain to 57.1% in New York City [4] after the first pandemic wave. A second living systematic review that focused on healthcare employee prevalence using only serology testing similarly found a wide range of prevalence across the studies (0% to 45.3% with an overall, pooled prevalence of 8.7%) [8]. The large variability can be explained by a variety of factors from their location and the outbreaks in the area, to measures both on the government level and on the level of the facilities, as well as on their kind of work, level of contact with high-risk persons, and novelty of the pandemic at that time. Knowledge of the prevalence in the general population is further necessary for understanding and comparing the additional risk and burden on this highly exposed population.

It is not fully understood what factors relating to behavioural, hygiene, exposure and environmental contribute to a potential increased risk in prevalence of SARS-CoV-2 infection in this population, and if it is different from those studied in the hospital setting or general population. It is also not understood what level of preventative measures and which specific preventative measures are most successful at reducing the spread of SARS-CoV-2 in nursing homes. While nurses in these facilities and those that provide at home care are at high risk of exposure due to their frequent and close contact with patients or residents, not much is understood about the differences in prevalence for staff members who provide other services in these facilities such as housekeeping, kitchen, transportation, administration and other services. One study of care homes from London found large variability in prevalence of their staff depending on level of exposure to residents as well as level of exposure to one or more care homes, however this study does not discern between roles of staff members [14]. Other studies in Switzerland have looked into these populations during the pandemic, but have focused on those staff members working in hospitals [5, 6, 15]. It is crucial to understand this knowledge gap of SARS-CoV-2 prevalence in a specific, non-hospital healthcare worker setting such as nursing homes and home healthcare.

With the current study, we aimed to gain a broader understanding of the risk and burden of nursing home and home healthcare employees who were potentially exposed to SARS-CoV-2 at their work during the first wave in Switzerland in spring 2020. Our specific aims were (1) to compare seroprevalence in these populations with the ones detected in the general population and to explore the proportion of seropositive people without COVID-19 attributable symptoms, (2) to assess factors associated with seropositivity and (3) to explore the experiences and attitudes of the employees as well as the facilitators and barriers for coping with the working situation during the SARS-CoV-2 pandemic. We aim for the results of this study to help inform future implementation of protection measures in these institutions.

## Methods

### Study design, setting and participants

This is a cross-sectional study which was conducted within the frame of the Corona Immunitas research program. Corona Immunitas encompasses over 40 cross-sectional and longitudinal seroprevalence studies and aims at determining the extent and nature of infection with SARS-CoV-2 in Switzerland in a consistent and comprehensive way [3].

The current sub-study targeted employees of selected nursing homes and home healthcare organizations (Spitex) in the canton of Zurich who were possibly exposed to SARS-CoV-2 at their work given there were positively tested nursing home residents or inhabitants of the communities, respectively, during the first wave in Switzerland between February and May/June 2020.

The strategy for the recruitment of the six nursing homes and six at home healthcare organizations is described in detail in the Additional file 1. In brief, nursing homes located in the canton of Zurich with the highest number of SARS-CoV-2 infected residents between February and May 2020, assessed by a survey of the nursing home association conducted in May 2020, were ordered in a sampling list according to the number of infected residents, starting with the highest number. The managing director of the nursing home association consecutively informed the nursing homes on the sampling list about the study and asked for participation. When the nursing homes agreed to participate, the Corona Immunitas project leader was informed who then directly contacted the directors of the nursing homes to plan the study procedures. The first six nursing homes contacted on the sampling list all agreed to study participation.

The at home healthcare organizations, namely Spitex organizations in the Canton of Zurich (www.spitexzh.ch) where trained staff provide care and support to people at home, were similarly recruited. Since home healthcare organizations are responsible for specific communities, we based the selection of the six home healthcare organizations on communities of the Canton of Zurich with the highest registered case numbers of SARS-CoV-2 infected inhabitants between February and June 2020. Out of the 238 communities in total, the Corona Immunitas project leader randomly allocated the specific organizations responsible for the 20 communities with the highest numbers to a sampling list and randomly selected six organizations. The managing director of the Spitex association Canton of Zurich then consecutively asked the selected organizations for study participation. If an organization refused participation, the next organization on the list was contacted. In case the organizations agreed, the managing director subsequently informed the Corona Immunitas project leader, who in turn directly contacted the particular healthcare organizations for planning of the assessments in the institution. In total, four organizations refused participation. All employees from the selected 12 organizations were invited to participate in the study. Assessments took place between 21st September and 23rd October 2020, before the second wave of the pandemic hit Switzerland.

These populations were compared to a population-based sample of randomly selected persons from the general population of the Canton of Zurich, stratified by age (20 to 64 years, above 65 years). This sampling was conducted by the Federal Statistical Office (FSO) of Switzerland. Blood drawing for serology of SARS-CoV-2 antibodies and questionnaires were assessed from the end of June to the beginning of September 2020 during phase two of the Corona Immunitas program [3]. Given that the retirement age in Switzerland is typically 64 or 65 years, we used the stratum of participants aged 20 to 64 from the Zurich population sample for comparability with the employee populations. All study participants provided written, informed consent prior to their participation in the study.

### Procedures and measurements

Recruitment was organized between the responsible persons from the organizations and the Corona Immunitas study staff. The organizations provided email addresses of interested employees, who were then sent by the study team the invitation for the study visit, the official document of the information for participants with the consent form and the link to the online baseline questionnaire implemented using the REDCap (Research Electronic Data Capture) database [16]. The participants also had the possibility to fill in the questionnaire later or on a paper/pencil version. These questionnaires are available in full in the appendix of the Corona Immunitas protocol paper [3]. The study visits were conducted at each of the 12 institutions where three to five trained Corona Immunitas staff members were present for 3–4 h, answered questions, took the participants’ written informed consent and drew 10 ml blood. Staff who could not attend the study visit in the organization was invited for the visit in the Epidemiology Biostatistics and Prevention Institute (EBPI) of the University of Zurich (less than 5% did).

The following information was collected by the *questionnaire*, in alignment with all Corona Immunitas studies: Participant characteristics: Age, sex, smoking status, nationality, highest education, number of persons in same household; health data: chronic conditions, body mass index (BMI), blood group, vaccination; COVID-19 specific information: episodes of symptoms yes/no, own positive real-time reverse transcription polymerase chain reaction (PCR) tests, positive PCR tests of persons in environment, use of SwissCovid App; risk behavior, exposure: adherence to general hygiene and physical distancing rules (including utilization of masks and gloves): number of times shopping, number of people met (reason for meeting people, reduction of people met since outbreak), travels abroad, level of concerns, health-related quality of life (EQ-5D-5L scale). In addition, the following occupational data specific to these populations was assessed: Work area, level of exposure to residents or clients, contact with SARS-CoV-2 infected resident or clients, protective measure knowledge, confidence, risk perception and attitudes. The occupational related portion also included three open-ended questions addressing the participants’ experiences with the biggest changes since the pandemic, of particular difficulties (barriers), and of what helped most (facilitators) in their daily work life during the pandemic (full questions provided in Additional file 1). Finally, we also addressed the directors of the six nursing homes and asked them to provide feedback to an open-ended question about specific difficulties faced in implementing the safety measures at the beginning of the pandemic.

### Laboratory analysis/serological testing

The antibody test used for the study was chosen by a team of experts from the Corona Immunitas consortium after extensive review. The Sensitive Anti-SARS-CoV-2 Spike Trimer Immunoglobulin Serological (SenASTrIS) assay developed by the Vaud Central University Hospital (CHUV), the Swiss Federal Institute of Technology in Lausanne (EPFL) and the Swiss Vaccine Center was chosen [17]. The test provides antibody results for IgG and IgA antibodies and has a specificity of 99.7% and high sensitivity of 96.6% post 15 days of infection and was used for the nursing home, home healthcare and Zurich population-based samples in this study. Presence of either IgG and/or IgA antibodies was counted as a positive test result.

### Statistical analysis

We used descriptive statistics to explore the characteristics of the study participants in comparison with the population-based sample and the perspective of employees regarding knowledge, confidence, risk perception and attitudes. The first aim of the study was the comparison of the seroprevalence of SARS-CoV-2 antibodies in the nursing home and home healthcare populations to that of the seroprevalence of the general population of Zurich between the first and second pandemic waves in Switzerland. The estimation of seroprevalence of the population-based sample was done using a Bayesian logistic regression model and adjusted with the sensitivity/specificity of the antibody test [18]. To assess factors associated with seropositivity, we reported the characteristics of the populations according to SARS-CoV-2 antibody presence. To assess the second aim, we used a multivariable logistic regression model to determine any associations based on what was found at the descriptive level, in order to determine if any behavioral factors contributed to the seroprevalence of these populations. The multivariable logistic regression model on prevalence included job role (nurse or other), job status (fulltime or part), smoking status, number of household members, SwissCovid app use, presence of symptoms, number of SARS-CoV-2 positive people in immediate environment and number of persons met outside household during the pandemic, to determine their effect on seroprevalence. These variables were chosen based on current evidence, mainly in the hospital setting, as well as what potential modifiable behaviors could have an impact on seroprevalence [5, 6]. We also performed checks to ensure no strong collinearity was found among the variables and no interaction terms were used in the model.

To assess the employees’ experiences as well as facilitators and barriers for coping with the working situation, we used conventional content analysis [19] with data-driven category development to analyze the three open-ended questions. Three team members (O.J.K., L.J.S., J.K) reviewed the answers independently, created labels for codes that reflected the key thoughts from the answers, defined descriptors and grouped them into categories. They agreed on this initial coding scheme and refined codes and categories. Then they grouped the categories into themes in a second round and sought additional agreement in the group. We described the numbers and percentages of persons who mentioned specific categories and themes in their answers. All analyses were conducted in R [20].

## Results

Baseline assessments were conducted between 21^st^ September and 23^rd^ October, corresponding to the beginning of the second pandemic wave in Switzerland and representative of the state after the first pandemic wave until the onset of the second due to delays in detectable seroconversion after infection. A total of 296 nursing home employees and 131 home healthcare employees were recruited into the study with participation rates of 39% (range 19–69%) and 25% (range 16–31%), respectively. The full descriptive statistics for the three distinct populations are shown in Table 1 and further descriptive statistics specific to the nursing home and home healthcare employees are shown in Table 2.

**Table 1:** Characteristics of the study participants and comparison with the population-based sample

	Home healthcare employees	Nursing homes employees	Population sample
Total participants	131	296	472
Age mean (sd)	44.7 (12.7)	43.5 (13.4)	44.7 (11.7)
Sex			
Female	122 (93.1%)	240 (81.1%)	246 (52.1%)
Male	9 (6.9%)	56 (19.0%)	226 (47.9%)
Smoking status			
Current smoker	34 (26.6%)	81 (29.6%)	117 (24.8%)
Ex-smoker	30 (23.4%)	49 (17.9%)	252 (53.5%)
Never smoker	64 (50.0%)	144 (52.6%)	102 (21.7%)
Citizenship			
Swiss	112 (85.5%)	201 (67.9%)	358 (75.8%)
Other	19 (14.5%)	96 (32.3%)	114 (24.2%)
Education			
Primary	6 (4.6%)	44 (14.9%)	13 (2.8%)
Secondary	68 (51.9%)	139 (47.0%)	185 (39.2%)
Tertiary	53 (40.5%)	88 (29.7%)	268 (56.8%)
Number of other people in the same household			
0	22 (17.2%)	43 (15.9%)	70 (15.0%)
1	43 (33.6%)	83 (30.7%)	176 (37.6%)
2 or more	63 (49.2%)	144 (53.3%)	144 (47.4%)
Chronic conditions			
No chronic condition	106 (82.8%)	217 (78.9%)	378 (80.3%)
At least one chronic condition^a^	22 (17.2%)	58 (21.1%)	93 (19.7%)
BMI mean (sd)	24.6 (5.0)	25.8 (4.7)	24.9 (4.9)
EQ-5D-5L dimensions			
Mobility problems present	7 (5.3%)	45 (15.2%)	45 (9.5%)
Problems with self-care present	3 (2.3%)	30 (10.1%)	5 (1.1%)
Problems during usual activities present	9 (6.9%)	33 (11.1%)	36 (7.6%)
Pain or discomfort present	43 (32.8%)	115 (38.7%)	134 (28.4%)
Anxiety or depression present	23 (17.6%)	83 (28.0%)	117 (24.8%)
EQ visual analogue scale (0 = worst; 100 = best) mean (sd)	86.6 (12.4)	85.7 (12.2)	85.3 (11.6)
Episodes of symptoms in 2020, at least for 3 days			
None	50 (38.2%)	141 (47.6%)	190 (40.3%)
1 or more	81 (61.8%)	155 (52.4%)	282 (59.7%)
Previous SARS-CoV-2 test results			
Tested positive for SARS-CoV-2	1 (0.8%)	18 (6.1%)	0 (0.0%)
Tested negative for SARS-Cov-2	42 (32.1%)	142 (48.0%)	41 (8.7%)
No test done	84 (64.1%)	126 (42.6%)	429 (91.1%)
Number of people in immediate environment previously tested positive for SARS-CoV-2			
0	117 (89.3%)	224 (75.4%)	424 (89.8%)
1	4 (3.1%)	14 (4.7%)	17 (3.6%)
2 or more	10 (7.6%)	59 (19.9%)	31 (6.6%)
Adherence to preventative measures in the last 7 days (1 = never; 5 = always), mean (sd)			
Keeping social distance	4.1 (0.8)	4.1 (1.0)	4.1 (0.7)
Only leaving house for essential tasks	3.0 (1.1)	3.7 (1.0)	3.2 (1.1)
Wearing face mask	4.2 (0.9)	4.5 (0.8)	3.0 (1.2)
Adhering to hygiene measures (washing hands, etc.)	4.7 (0.5)	4.7 (0.5)	4.6 (0.6)
Use of SwissCovid App			
Yes, regularly	42 (32.8%)	69 (25.4%)	203 (53.7%)
No	80 (62.5%)	187 (68.8%)	147 (38.9%)
Number of trips taken outside the country since January 2020			
0	53 (40.5%)	125 (42.2%)	251 (53.2%)
1	42 (32.1%)	69 (23.3%)	110 (23.3%)
2 or more	36 (27.5%)	102 (34.5%)	111 (23.5%)

All values are shown as n (%) unless otherwise noted

*sd* standard deviation; *BMI* Body Mass Index; *COVID-19* Coronavirus Disease 2019; *EQ* EuroQol; *DFU* Digital Follow Up; *PCR* Reverse Transcription Polymerase Chain Reaction Test; *SARS-CoV-2* Severe Acute Respiratory Syndrome Coronavirus 2 Infection

^a^Excluding allergies

**Table 2:** Nursing home and home healthcare employees specific characteristics

	Home healthcare employees (n = 131)	Nursing home employees (n = 296)
Job function		
Nurse, care	92 (71.9%)	155 (57.4%)
Housekeeping, kitchen and technical services	9 (7.0%)	78 (28.9%)
Administration	16 (12.5%)	24 (8.9%)
Other	11 (8.6%)	13 (4.8%)
Work status		
Part-time (< 80%)	73 (55.7%)	70 (23.6%)
Full-time (80–100%)	58 (44.3%)	227 (76.4%)
Direct contact with residents/clients at work		
To a great extent or always	16 (12.5%)	60 (22.1%)
Occasionally	94 (73.4%)	204 (75.3%)
None or next to none	18 (14.1%)	7 (2.6%)
Unprotected close contact to residents/clients during pandemic		
Yes, any	71 (55.5%)	133 (49.8%)
No, none	57 (44.5%)	134 (50.2%)
Care of SARS-CoV-2 positive residents/clients		
Yes, currently	0 (0.0%)	8 (3.0%)
Yes, in the past	13 (10.2%)	84 (31.3%)
No or don’t know	115 (89.8%)	176 (65.7%)
Self-assessed knowledge (1 = not at all; 10 = fully agree), mean (sd)		
Knowing the transmission routes of SARS-CoV-2	9.0 (12.4)	9.0 (13.7)
Knowing how to use protective materials for work	9.5 (7.1)	9.6 (7.9)
Knowing how to recognize Covid-19 symptoms and suspicious cases	9.0 (12.9)	9.0 (13.6)
Knowing the steps to take if a resident/client has Covid-19	9.1 (14.3)	9.2 (14.6)
Assessment of confidence, risk perception, burden (1 = not at all; 10 = fully confident/likely/serious/burdensome), mean (sd)		
Confidence to protect oneself at work from SARS-CoV-2 infection	7.9 (18.2)	7.8 (19.6)
Confidence to protect residents/clients from SARS-CoV-2 infection	7.8 (18.6)	7.5 (22.0)
Likelihood of contracting the coronavirus at work	4.3 (29.0)	4.4 (26.0)
Likelihood of infecting a resident with the coronavirus		5.4 (25.6)
Extent of seriousness of infection with the coronavirus personally	4.2 (26.5)	4.7 (26.8)
Burden of protective measures at work for oneself		5.7 (30.4)
Concern of persons in private environment that you work in the health sector	5.1 (32.4)	4.8 (31.0)

All values are shown as n (%) unless otherwise noted

*sd* standard deviation; *SARS-CoV-2* severe acute respiratory syndrome coronavirus 2 infection

The nursing home staff comprised of 81% women with an average age of 43.5 years. 57% work as nurses and care support and the remainder work in general housekeeping, cleaning, kitchen and technical services (29%), administration (9%), and other miscellaneous functions (5%). The home healthcare employees comprised of 93% women with an average age of 44.7 years. 72% work as nurses and care support and the remainder work in administration (13%), general housekeeping and cleaning (7%) and other miscellaneous functions (8%). Home healthcare employees reported less frequent or no direct contact with residents/clients (14.1%) at work compared to the nursing home staff (2.6%). The self-assessed knowledge on SARS-CoV-2 and Covid-19 specific information was very high (> 9 out of 10 points) and did not differ between the two groups. Confidence to protect residents/clients and oneself was high (> 7 out of 10 points), and assessed likelihood to contract residents/clients and oneself was moderate (> 4, < 6 out of 10 points).

For the general population of the Canton of Zurich, the overall SARS-CoV-2 seroprevalence for those age 20–64 was 3.5% (95% CI 1.5% to 5.9%) (Table 3). SARS-CoV-2 seroprevalence for nursing home employees was much higher at 14.9% (95% CI 11.1–19.6%; range 3.8% to 24.4%), and the prevalence of home healthcare employees was more similar to the general population at 3.8% (95% CI 1.4% to 9.1%; range 0% to 10%) (Table 4).

**Table 3:** SARS-CoV-2 antibody prevalence by population

	Nursing home & home healthcare populations			Population sample		
	Total N	No presence of antibodies	Presence of antibodies	Total N	No presence of antibodies	Presence of antibodies
Total participants	427	378	49 (11.5%)	472	453	19 (3.5%^a^)
Asymptomatic (since January 2020)	49	40	9 (18.4%)	18	14	4 (22.2%)
Age mean (sd)	43.8 (13.2)	43.7 (13.1)	45.4 (13.9)	44.7 (11.7)	44.6 (11.7)	46.2 (11.0)
Sex						
Female	361	319	42 (11.6%)	246	239	7 (2.8%)
Male	65	59	6 (9.2%)	226	214	12 (5.3%)
Smoking status						
Current smoker	114	108	6 (5.3%)	102	95	7 (6.9%)
Ex-smoker	79	69	10 (12.7%)	117	112	5 (4.3%)
Never smoker	208	179	29 (13.9%)	252	245	7 (2.8%)
Citizenship						
Swiss	313	276	37 (11.8%)	358	342	16 (4.5%)
Other	115	101	12 (10.6%)	114	111	3 (2.6%)
Education						
Primary	49	40	9 (18.4%)	13	12	1 (7.7%)
Secondary	206	184	22 (10.7%)	185	179	6 (3.2%)
Tertiary	140	128	12 (8.6%)	268	256	12 (4.5%)
Number of other people in the same household						
0	64	58	6 (9.4%)	70	68	2 (2.9%)
1	125	116	9 (7.2%)	176	167	9 (5.1%)
2 or more	206	180	26 (12.6%)	222	214	8 (3.6%)
Chronic conditions						
No chronic condition	321	286	35 (10.1%)	378	364	14 (3.7%)
1 or more chronic conditions^b^	79	70	9 (11.4%)	93	88	5 (5.4%)
BMI (mean, sd)	25.4 (4.8)	25.3 (4.8)	26.0 (5.0)	24.9 (4.9)	25.0 (4.9)	23.1 (3.8)
EQ—5D-5L dimensions						
Mobility problems present	52	45	7 (13.5%)	45	42	3 (6.7%)
Problems with self-care present	33	27	6 (18.2%)	5	5	0 (0.0%)
Problems during usual activities present	42	36	6 (14.3%)	36	35	1 (2.9%)
Pain or discomfort present	157	141	16 (10.2%)	134	128	6 (4.5%)
Anxiety or depression present	106	98	8 (7.6%)	117	109	8 (6.8%)
EQ visual analogue scale (0 = worst; 100 = best) mean (sd)	86.0 (12.3)	85.9 (12.4)	86.4 (11.4)	85.3 (11.6)	85.2 (11.6)	86.3 (11.8)
Episodes of symptoms in 2020, at least for 3 days						
No	194	179	15 (7.7%)	190	185	5 (2.6%)
1 or more	233	199	34 (14.6%)	282	268	14 (5.2%)
Previous SARS-CoV-2 test results						
Tested positive for SARS-CoV-2	18	3	15 (83.3%)	0	0	0 (0.0%)
Tested negative for SARS-CoV-2	163	150	13 (8.0%)	40	38	2 (5.0%)
No test done	208	194	14 (6.7%)	429	412	17 (4.0%)
Number of people in immediate environment previously tested positive for SARS-CoV-2						
0	339	308	31 (9.1%)	424	407	17 (4.0%)
1	18	15	3 (16.7%)	17	16	1 (5.9%)
2 or more	68	54	14 (20.6%)	31	30	1 (3.2%)
Adherence to preventative measures in the last 7 days (1 = never; 5 = always), mean (sd)						
Keeping social distance	4.1 (0.9)	4.1 (0.9)	4.2 (0.9)	4.1 (0.7)	4.1 (0.8)	4.2 (0.5)
Only leaving house for essential tasks	3.4 (1.1)	3.4 (1.1)	3.6 (0.9)	3.2 (1.1)	3.2 (1.1)	3.3 (1.1)
Wearing face mask	4.4 (0.8)	4.4 (0.8)	4.4 (0.7)	3.0 (1.2)	3.0 (1.2)	2.9 (1.1)
Adherence to hygiene measures (washing hands, etc.)	4.7 (0.5)	4.7 (0.5)	4.6 (0.7)	4.6 (0.6)	4.6 (0.6)	4.6 (0.6)
Use of SwissCovid App						
Yes, regularly	133	123	10 (7.5%)	231	220	11 (4.8%)
No	266	231	35 (13.2%)	147	140	7 (4.8%)
Number of trips taken outside the country since January 2020						
0	178	158	20 (11.2%)	251	242	9 (3.6%)
1	110	100	10 (9.1%)	110	107	3 (2.7%)
2 or more	136	118	18 (13.2%)	111	104	7 (6.3%)

All values are shown as n or n (%) unless otherwise noted

*sd* standard deviation; *BMI* Body Mass Index; *COVID-19* Coronavirus Disease 2019; *DFU* Digital Follow Up; *PCR* Reverse Transcription Polymerase Chain Reaction Test; *SARS-CoV-2* Severe Acute Respiratory Syndrome Coronavirus 2 Infection

^a^Prevalence estimates for population sample were calculated using a Bayesian logistic regression model and weighted for age, sex and sensitivity/specificity of the antibody test

^b^Excluding allergies

**Table 4:** SARS-CoV-2 antibody prevalence for nursing home and home healthcare specific characteristics

	Total N	No presence of antibodies	Presence of antibodies
Home healthcare	131	126	5 (3.8%)
Nursing homes	296	252	44 (14.9%)
*Job function*			
Nurse, care	247	211	36 (14.6%)
Housekeeping, kitchen and technical services	86	79	7 (8.1%)
Administration	40	39	1 (2.5%)
Other	24	23	1 (4.2%)
Work status			
Part-time (< 80%)	143	131	12 (8.4%)
Full-time (80–100%)	282	246	36 (12.8%)
Direct contact with residents/clients at work			
To a great extent or always	76	69	7 (9.2%)
Occasionally	297	260	37 (12.5%)
None or next to none	25	24	1 (4%)
Unprotected close contact to residents/clients during pandemic			
Yes, any	201	179	22 (10.9%)
No, none	191	169	22 (11.5%)
Care of SARS-CoV-2 positive residents/clients			
Yes, currently	8	6	2 (25.0%)
Yes, in the past	97	80	17 (17.5%)
No or don’t know	288	264	24 (8.3%)
Self-assessed knowledge (1 = not at all; 10 = fully agree), mean (sd)			
Knowing the transmission routes of SARS-CoV-2	9.0 (13.3)	9.0 (13.2)	9.0 (14.1)
Knowing how to use protective materials for work	9.5 (7.7)	9.5 (7.5)	9.6 (8.7)
Knowing how to recognize Covid-19 symptoms and suspicious cases	9.0 (13.4)	9.0 (13.8)	9.2 (10.0)
Knowing the steps to take if a resident/client has Covid-19	9.1 (14.5)	9.1 (14.8)	9.3 (12.5)
Assessment of confidence, risk perception, burden (1 = not at all; 10 = fully confident/likely/serious/burdensome), mean (sd)			
Confidence to protect oneself at work from SARS-CoV-2 infection	7.8 (19.2)	7.8 (19.4)	7.9 (17.6)
Confidence to protect residents/clients from SARS-CoV-2 infection	7.6 (21.0)	7.7 (20.4)	7.2 (25.4)
Likelihood of contracting the coronavirus at work	4.4 (27.0)	4.3 (26.8)	4.6 (28.6)
Likelihood of infecting a resident with the coronavirus	5.4 (25.5)	5.4 (25.5)	5.4 (26.5)
Extent of seriousness of infection with the coronavirus personally	4.5 (26.8)	4.5 (26.8)	4.7 (26.8)
Burden of protective measures at work for oneself	5.7 (30.3)	5.8 (29.6)	5.3 (33.6)
Concern of persons in private environment that you work in the health sector	4.9 (31.5)	4.9 (31.1)	4.7 (34.5)

All values are shown as n or n (%) unless otherwise noted

*sd* standard deviation; *SARS-CoV-2* severe acute respiratory syndrome coronavirus 2 infection

^a^Prevalence estimates for population sample were calculated using a Bayesian logistic regression model and weighted for age, sex and sensitivity/specificity of the

antibody test

**Fig. 1:**
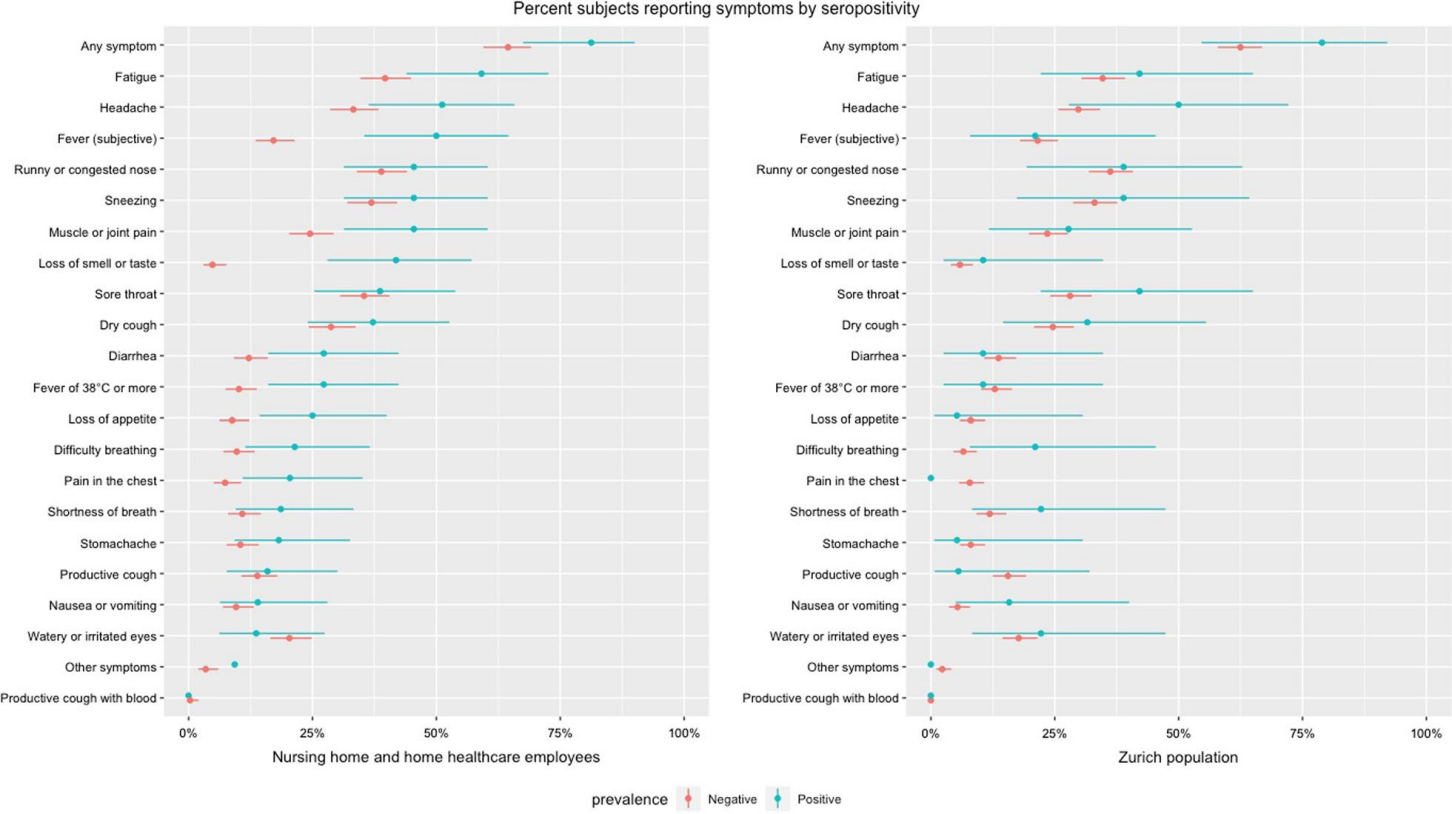
Percent subjects reporting symptoms by seropositivity

Of the total 49 participants with the presence of IgG or IgA antibodies in the combined nursing home and home healthcare populations, 9 (18.4%) reported having no COVID-19 related symptoms since January 2020 compared to 22.2% in the general population of Zurich who were seropositive. Symptoms and their frequencies were reported in Fig. 1. The most frequently reported symptom of those in the home healthcare/nursing home populations was fatigue, regardless of antibody status. 42.3% of employees reported fatigue symptoms, 59.1% of those who were seropositive and 39.7% of those who were seronegative. While fatigue was also commonly reported in the Zurich population (42.1% of the seropositives and 34.7% of the seronegatives), the most frequently reported symptoms were headache for those who were seropositive (50.0%) and runny or congested nose for those who were seronegative (36.2%).

Some other differences were found between those nursing home and home healthcare employees who were seropositive for SARS-CoV-2 and those who were not (Table 3). Seropositive employees were slightly older, with a mean age of 45.4 compared to 43.7. 5.3% of current smokers were seropositive, while 12.7% and 13.9% of ex- and never smokers were seropositive, respectively. Those participants who reported use of the SwissCovid App provided by the Swiss Federal Office of Public Health (FOPH) [21] had a lower rate of seropositivity (7.5%) compared to those who did not use the app (13.2%). Those employees who reported some or frequent contact with residents and clients had higher rates of seropositivity than those employees who did not (12.5%, 9.2%, 4.0%, respectively).

The odds ratios (OR) and 95% confidence intervals of the multivariable model of seroprevalence are reported in Table 5. It found that nurses were significantly more likely to have SARS-CoV-2 antibodies than those employees who were not nurses (OR 2.606; 95% CI 1.097–6.189). Number of persons met outside the household (OR 0.920; 95% CI 0.872–0.971), whether they had symptoms (OR 2.740; 95% CI 1.096–6.847), and current smoking status (OR 0.260; 95% CI 0.097–0.696) were similarly significantly associated with seroprevalence. The population of Zurich was not included in the model as the intention was to determine if any behavioral or sociodemographic factors had an impact on prevalence in the nursing home and home healthcare worker populations.

**Table 5:** Multivariable logistic regression of prevalence in nursing home and home healthcare workers

Variables	SARS-CoV-2 prevalence	
	Odds ratio	95% Confidence intervals
Nurse	2.606	1.097–6.189
Fulltime work	1.250	0.565–2.763
Current smoker	0.260	0.097–0.696
Ex-smoker	0.583	0.207–1.641
Number of household members	1.297	0.988–1.703
SwissCovid app use	0.592	0.251–1.397
Symptomatic	2.740	1.096–6.847
Number of SARS-CoV-2 positive people in immediate environment	1.219	0.925–1.606
Number of persons met outside household during pandemic	0.920	0.872–0.971

Pseudo R = 0.1809

The three open-ended questions asked the employees about the biggest change since the pandemic, particular difficulties (barriers), and what helped (facilitators) in their daily work life during the pandemic. 207, 205 and 187 out of 296 nursing home employees and 109, 95 and 87 out of 131 home healthcare employees (Table 6 with further details in the Additional file 1: Tables S1–S3) responded, respectively. There were no major differences between the two professional groups. The implementation of general safety measures was viewed as the most crucial change (n = 133, 61.3%, n = 75, 68.8%) that further introduced both emotional distance and lack of closeness to the residents/clients and colleagues, as well as the practical burden of wearing protective clothes and hand hygiene. Since issues relating to face masks were very frequently and specifically mentioned by the employees (n = 109, 50.2%, n = 75, 68.6%), we categorized issues around wearing protective masks into an additional category. Most crucial for the employees were the obligation to wear it constantly and its negative impact on mimicry and communication with the residents/clients. Wearing face masks was also the most frequently answered category to the question on the biggest barriers in daily work by both groups (n = 139, 67.8%, n = 62, 65%), followed by psychosocial aspects (n = 78, 38.1%, n = 27, 33.7%) where they mentioned the uncertainty and fear of spreading the virus to residents/clients, the concern for residents and dealing with relatives/visitors. Work-related changes (n = 21, 10.2%, n = 6, 6.3%) and general safety measures were seen as less important.

**Table 6:** Summarized overview of themes developed from answers to open-ended questions

Themes	Nursing home employees n (%)	Home healthcare employees n (%)
What was the biggest change in your daily work life since the start of the coronavirus pandemic?		
Total of answers	217/296 (73.1)	109/131 (83.2)
General safety measures (except for mask use)	133 (61.3)	75 (68.8)
Wearing hygiene face mask	109 (50.2)	75 (68.8)
Work-related changes	77 (33.1)	13 (11.9)
Psychological aspects	45 (20.3)	24 (22)
Other	17 (8.3)	12 (11)
What is currently particularly difficult for you in your daily work life?		
Total of answers	205/296 (69.0)	90/131 (72.5)
Wearing hygiene face mask	139 (67.8)	62 (65.3)
Psychosocial aspects	78 (38.1)	27 (33.7)
Work-related changes	21 (10.2)	6 (6.3)
General safety measures (except for mask use)	20 (9.8)	10 (10.5)
Other	26 (9.3)	15 (15.8)
What helps you to conduct your daily work under the current conditions?		
Total of answers	187/296 (63.0)	87/131 (66.4)
Psychosocial aspects	126 (67.4)	54 (62.1)
Work-related changes	48 (25.7)	27 (31.1)
Safety measures (except for mask)	30 (16.0)	22 (25.3)
Other	17 (9.1)	20 (23.0)

The greatest facilitators to cope with the current situation were psychosocial aspects (n = 126, 67.4%, n = 54, 62.1%). The employees found that team spirit, including a good working atmosphere and support and solidarity between colleagues, and emotional coping (such as humor, positivity, and self-confidence), and problem-oriented coping (such as setting priorities and discipline) were particularly helpful during the pandemic. Some work-related changes were also reported to be helpful (n = 48, 25.7%, n = 27, 31.1%), particularly management providing clear guidelines, knowledge transfer on protection concepts and support from superiors, job security and practical issues such as breaks. Finally, the safety measures were also perceived to be helpful (n = 30, 16%; n = 22, 25.3%).

According to the nursing home directors’ feedback, half of the nursing homes (n = 3) experienced a shortage in protection material (masks, disinfection, and protective gear) at the pandemic onset. Other challenges were insecurity among residents and employees due to increasing fear of deaths (n = 2), the great burden for residents because of the visiting ban (n = 1), frequently changing official regulations for implementing protective measures (n = 1), and that residents and relatives did not understand why they needed to follow measures and often did not do so (n = 1).

## Discussion

Our results suggest that nursing home employees had higher rates of SARS-CoV-2 infection compared to the general population of the Canton of Zurich, while home healthcare employees had prevalence rates more similar to that of the general population. For both professional groups, the implementation of general safety measures, especially the constant wearing of the face mask, were perceived as the most crucial and difficult change in their daily work, whereas team spirit and solidarity between colleagues as well as personal emotional coping with the situation were perceived to be the most important facilitators to help with their work life during the pandemic.

Prevalence rates for healthcare employees were shown in a systematic review to be 8.5% for the Europe region and was found to be 9.6% and 3.0% in two other Swiss studies [6, 8, 15], all of which are slightly lower than the prevalence found in our nursing home employee population. The higher overall prevalence rate in nursing home employees reflects our target population of employees of nursing homes which experienced outbreaks and the resulting higher probability of exposure to a SARS-CoV-2 infected residents or colleagues. Although we selected home healthcare organizations based on the extent of SARS-CoV-2 infection rates within their communities, the SARS-CoV-2 prevalence in the Canton of Zurich was low in spring 2020, and so was the likelihood that infected persons were concurrently clients of the home health care organizations. In addition, home healthcare employees have usually shorter contact times with clients compared to the nursing home population with residents, and they tended to more frequently work part-time. Therefore, the overall likelihood of coming into contact with infected SARS-CoV-2 patients and time spent with them was most likely higher for nursing home employees.

However, these factors do not explain the difference in prevalence rates between the six different nursing homes that range from 3.8 to 23.8%. The nursing home directors’ input similarly could not shed light on this. Both, the nursing home director from the nursing home with the lowest and with the highest prevalence rate, reported that shortage in protection material was one of the biggest challenges at pandemic onset. Not being able to implement best possible protection measures due to this shortage was mentioned by some directors; however, it was not a reported issue for three out of the six nursing home directors. Except for one director from a nursing home with an average infection rate who reported not having experienced specific challenges, the directors experienced the time at pandemic onset as burdensome due to insecurity over how to handle this new situation, implementation of visiting bans for residents and frequent changes of official regulations on the use of protective measures.

Overall, there was no obvious pattern apparent between those with low and high prevalence rates. One could only speculate that at pandemic onset when there was still no or little knowledge available about the virus and its transmission and no protection measures implemented yet, it might also just be a random result into which institution the virus was brought and transmitted or not.

The main results from the multivariable analysis showed that nurses were more likely to have antibodies than those employees who were in administration or other roles. The use of the SwissCovid app was associated with less risk of SARS-CoV-2, however it was not significant in a multivariable model. In both the multivariable model and Table 3, it is shown that current and ex-smokers are less likely to have antibodies than never smokers only in our nursing home and home healthcare employee populations which is in line with the current literature that smokers seem to produce less, if any, antibodies and/or have antibody responses that wane faster than their non-smoking counterparts [23]. Many of the behaviors relating to protective measures and the knowledge relating to SARS-CoV-2 and how to protect against it were not associated with prevalence when assessed univariably, however the participants across all populations tended to report the same level of knowledge and behavioral implementations. Our results are comparable to the literature on healthcare employees during the SARS-CoV-2 pandemic. It is frequently shown that those employees in the healthcare sector that are front line with patients are at much higher risk of infection, compared to those with more administrative, food/housekeeping, and technical roles [8]. Furthermore, the shortage of equipment, particularly at the beginning of the pandemic was also reported consistently in the literature to be one of the factors associated with excess risk of exposure and potentially avoidable deaths [24] and was similarly mentioned by the directors of the nursing homes to be a major issue they faced during the beginning of the pandemic.

The SARS-CoV-2 pandemic introduced changes for the employees in their daily work, especially due to the preventive measures. Similarly, the implementation of the preventive measures in their institution was challenging for the directors. For coping with similar situations in the future, the organization will generally profit from the experiences they made and processes they established during this period. Since one of the main problems was adhering to the general safety measures, pandemic training could prepare staff for the situation in advance and thus reduce the stress factor of the unknown.

One main limitation the employees experienced was wearing face masks the entire day. Providing the staff with transparent masks, if affordable in the future, would facilitate the communication with the residents and clients. Additionally, many personal problems associated with wearing masks mentioned by the participants as skin irritations could be reduced by supplying enough face masks to allow them to change frequently or by providing allergy-friendly masks. The physical and emotional distance to other people, reported as one of the most challenging changes, could be reduced by having staff members carry pictures of themselves without masks to re-establish a certain emotional closeness. Some possibilities to mitigate the burdensome perception of the measures would be to closely involve the employees in the concrete implementation of measures and to promote team spirit. Team spirit could be improved through regular meetings in small groups, and regular breaks could lead to better well-being.

The main strength of our study is that we compared our nursing home and home healthcare employee samples with a population-based, representative sample of their respective Cantonal populations and that the same rigorously assessed measurements were used for all samples, i.e. the serology test and questionnaires were consistent across all sites and populations. The self-reported data provided by the questionnaires help to provide more in depth understanding of transmission dynamics in the workplace and the effect of socioeconomic characteristics.

One limitation of our study is that the participation rate between the institutions differed a lot and, in some institutions—particularly in some home healthcare organizations, it was low. One explanation could be that, because we were present at the institutions only during a few hours and many persons work part-time, a considerable proportion of the employees were not on-site during the assessments and randomly did not participate. Moreover, for home healthcare employees, it is possible that those persons who worked at the assessment day might just have been at a client’s home visit at this time, which further explains the higher participation of nursing home employees. However, we cannot exclude the possibility that selected participation has happened in the different organizations, especially in at home healthcare employees, which could have introduced selection bias. We speculate that the risk for selection bias was lower for the selection of nursing home employees which is reflected by highly variable prevalence rates found in other studies [22].

According to our experience, home healthcare employees and organizations were more reluctant to participate in the study and only half of the asked home healthcare organizations participated compared to all asked nursing homes. The reasons they provided included skepticism towards studies in general, reluctance over providing blood samples and overall mixed-feelings about the pandemic. We do not have an assumption whether persons who were more prone to SARS-CoV-2 infection more or less frequently participated, i.e. in what direction the selection bias might have acted. Another limitation is that not all of those who are infected with SARS-CoV-2 necessarily develop antibodies to fight the infection [13] and that antibodies could have faded between time of infection and time of study participation [25]; therefore, it is possible that the overall prevalence is underestimated. However, since we used a comparison group of the general population of Zurich and consistent testing across the populations, the comparison between groups is accurate—thus showing a higher burden of infection for those nursing home staff members compared to the other populations.

## Conclusions

Our study results suggest that nursing home employees were more affected by SARS-CoV-2 infection compared to the general population and to home healthcare employees. However, no clear patterns were found regarding potential risk factors. The global pandemic introduced major changes for the employees in their daily work, especially due to the preventive measures and the requirement of wearing a face mask the entire day. However, personal resources and team support were particularly important resources to cope with the burdensome changes. To handle similar situations in the future, organization will profit from these learned experiences from this pandemic.

## Supplementary Information

**Additional file 1. Supplemental Tables S1–S3.** Detailed information on recruitment of organizations and the research group of Corona Immunitas.

## Data Availability

The datasets used and analysed during this study are available from the corresponding author on reasonable request.

## Abbreviations

BMI: Body Mass Index
CHUV: Vaud Central University Hospital
COVID-19: Coronavirus disease 2019
EBPI: Epidemiology, Biostatistics and Prevention Institute of the University of Zurich
EPFL: Swiss Federal Institute of Technology in Lausanne
FOPH: Swiss Federal Office of Public Health
FSO: Swiss Federal Office of Statistics
REDCap: Research Electronic Data Capture
RT-PCR a/k/a PCR: Reverse Transcription Polymerase Chain Reaction Test
OR: Odds Ratio
SARS-CoV-2: Severe Acute Respiratory Syndrome Coronavirus 2 Infection
SenASTrIS: Sensitive Anti-SARS-CoV-2 Spike Trimer Immunoglobulin Serological
