# Supplementary material for: Seroprevalence of SARS-CoV-2 antibodies, associated factors, experiences and attitudes of nursing home and home healthcare employees in Switzerland
Source: BMC Infect Dis. 2022 Mar 16;22:259. doi: 10.1186/s12879-022-07222-8 (PMC8924739; doi:10.1186/s12879-022-07222-8)
Supplement: Supplementary file 1 — Additional file 1. Supplemental Tables S1–S3. Detailed information on recruitment of organizations and the research group of Corona Immunitas. [file 12879_2022_7222_MOESM1_ESM.docx]

**Supplementary material**

**Detailed information on recruitment of organizations**

**Nursing homes**

For the recruitment of the nursing homes, the Corona Immunitas project leader collaborated with the manageing director of the nursing home association ([www.curaviva.ch](http://www.curaviva.ch)). We aimed to select six nursing homes in the Canton of Zurich that had residents with confirmed infection of SARS-CoV-2 between February and May 2020. The number of infected residents per organization was assessed by a survey sent by the nursing home association on 18 May 2020 to the 281 nursing homes, and they had time until the end of May to complete and return the survey. Based on these results, 22 out of the 124 nursing homes who answered the survey reported positively tested residents. We did not consider those five nursing homes for sampling with less than 50 employees. Out of the 17 remaining nursing homes, those with the highest number of infected residents were consecutively informed and asked for study participation from the nursing home association. In case of agreement, the nursing home association informed the Corona Immunitas project leader who directly contacted the nursing home directors to plan the further procedures. The first six institutions that were contacted agreed to participation in the study.

**Home healthcare organizations**

The at home healthcare organizations of trained staff providing care and support to people at home (Spitex organizations in the Canton of Zurich; [www.spitexzh.ch](http://www.spitexzh.ch)) were similarly recruited. Since single home healthcare organizations are responsible for their specific communities, we based the selection of the six home healthcare organizations on communities in the Canton of Zurich and aimed to recruit those organizations which were responsible for the communities with the highest amount of SARS-CoV-2 infected inhabitants between February and June 2020. A list including registered case numbers per community was provided by the Canton of Zurich’s Health Department (<https://www.zh.ch/en/gesundheitsdirektion.html>). Out of 238 communities in total, we selected in a first step those communities with the highest number of infections considering the number of inhabitants using the criteria “number of infections per community >=15” AND “number of infections per 10,000 inhabitants >=22”, which resulted in 43 communities. From those 43 communities, we did not consider thirteen communities where the high number of infections were caused by infected nursing home residents in these communities who are not possible clients from the community. Furthermore, we did not consider ten communities because the responsible organizations had less than 50 employees. From the remaining 20 communities, the responsible home healthcare organizations were assigned to three groups: (1) urban organizations (n=10); (2) larger rural organizations with >100 employees (n=4); and (3) smaller rural organizations with <100 employees (n=6). In each group, the Corona Immunitas project leader randomly allocated the organizations to a sampling order. The managing director of the Spitex association Canton of Zurich then consecutively asked the organizations for study participation. In case the organizations agreed, she subsequently informed the Corona Immunitas project leader, who in turn directly contacted the particular healthcare organizations for concrete planning of the assessments in the institution. If an organization refused participation, the next organization on the list was asked for participation. In the three groups, one urban organization, two larger rural organizations and one smaller rural organization refused participation.

**Overview on categories and themes developed of answers to open-ended questions**

**Table S1: What was the biggest change in your daily work since the outbreak of the corona epidemic?**

| **Nursing homes employees, n=217 (out of 296, 73.1%)** | | | **Home healthcare organization employees, n=109 (out of 131, 83.2%)** | | |
| --- | --- | --- | --- | --- | --- |
| **Themes** | **Categories** | **Descriptors** | **Themes** | **Categories** | **Descriptors** |
| **General safety measures (except for mask)**  *N=133  (61.3%)* | Distance/barrier to other people  *N=58 (43.6%)* | To residents or staff to comply with the safety measures, but also has an emotional aspect like lack of closeness to other human beings | **General safety measures (except for mask)**  *N=75 (68.8%)* | Distance/barrier to other people  *N=32 (42.7%)* | To clients or staff, to comply with the safety measures, but also has an emotional aspect like lack of closeness to other human beings |
|  | Adhere to hygiene measures *N=39 (29.3%)* | Adhere to protective measures, protective concept, or protective clothing |  | Adhere to hygiene measures  *N=14 (18.7%)* | Adhere to protective measures, protective concept or protective clothing |
|  | The hand hygiene  *N=20 (15%)* | Handy hygiene in general, hand disinfection and hand washing |  | No handshaking  *N=13 (17.3%)* | With the clients |
|  | Isolation of residents *N=9 (6.8%)* | Due to Covid-19 infection or symptoms |  | The hand hygiene  *N=11 (14.7%)* | Handy hygiene in general, hand disinfection and hand washing |
|  | No handshaking  *N=4 (3%)* | With the residents or their relatives |  | Disinfecting in general  *N=3 (4%)* | Disinfecting work surfaces or work areas after use |
|  | Disinfecting in general *N=3 (2.3%)* | Work surfaces or work areas after use |  | Isolation of clients *N=2 (2.7%)* | Due to Covid-19 infection or symptoms |
| **Wearing hygiene face mask**  *N=109  (50.2%)* | Wearing masks during work  *N=76 (69.7%)* | In the office or at work with the residents | **Wearing hygiene face mask**  *N=75 (68.8%)* | Wearing masks during work  *N =46 (61.3%)* | In the office or at work with clients |
|  | Mask obligation  *N=22 (20.2%)* | When participants especially mentioned the obligation of wearing a mask |  | Mask obligation during work  *N=11 (14.7%)* | When participants especially mentioned the obligation of wearing a mask |
|  | Limited communication  *N=7 (6.4%)* | No lip reading, problem for residents with hearing problems |  | Limited communication  *N=8 (10.7%)* | No lip reading, the understanding is acoustically worse, problem for clients with hearing problems |
|  | Limited mimic  *N=4 (3.7%)* | Mimic is not visible for residents and therefore it is irritating for them |  | Limited mimic  *N=7 (9.3%)* | Mimic is not visible for clients which irritates them |
|  |  |  |  | Other  *N=3 (4%)* | No air, sweating under the mask, throat irritation |
| **Work related changes**  *N=77  (33.1%)* | More workload  *N=32 (41.5%)* | Administrative but also with the residents. Due to safety and precautionary measures or temperature measurement of the residents | **Work related changes**  *N=13  (11.9%)* | More workload  *N=7 (53.8%)* | Administrative but also with the clients due to safety and precautionary measures |
|  | Changed work processes  *N=15 (19.5%)* | More complicated and complex processes |  | Home office  *N=4 (30.8%)* | Not working from the office but from home |
|  | Visiting ban/regulated visiting times  *N=7 (9.1%)* | To the nursing home for relatives and other visitors |  | Changed working atmosphere  *N=2 (15.4%)* | Light-heartedness is gone |
|  | Changed working atmosphere  *N=5 (6.5%)* | Mostly that the light-heartedness is gone |  |  |  |
|  | Adhere to regulations  *N=4 (5.2%)* | Makes work more complicated and exhausting |  |  |  |
|  | More flexibility needed *N=4 (5.2%)* | Work shift changed due to quarantine or isolation of staff |  |  |  |
|  | No events/excursions  *N=4 (5.2%)* | Are not allowed to take place due to restrictions |  |  |  |
|  | Staff absences  *N=4 (5.2%)* | Due to quarantine or isolation of staff members |  |  |  |
|  | Restaurants closed  *N=2 (2.6%)* | Due to visiting restrictions |  |  |  |
| **Psychological aspects**  *N=45 (20.3%)* | Increased awareness on safety and hygiene measures *N=7 (15.6%)* | Being aware of them and comply to them makes the daily work more challenging | **Psychosocial aspects**  *N=24  (22%)* | Reduced team contact  *N=6 (25%)* | With staff during breaks or at lunch |
|  | More insecurity  *N=6 (13.3%)* | Because of the constantly changing circumstances due to the pandemic |  | Clients suffer due to current situation  *N=5 (20.8%)* | Because of the regulations, the social distancing and the restrictions |
|  | Changes during leisure  *N=6 (13.3%)* | Increased awareness also at home, to not bring the virus to work and infect residents |  | Changed client contact  *N=4 (16.7%)* | Less contact and no affection allowed which results in a more distant contact |
|  | Focus on pandemic  *N=5 (11.1%)* | All attention on the pandemic |  | More insecurity  *N=3 (12.5%)* | Because of the constantly changing circumstances due to the pandemic |
|  | More fear  *N=5 (11.1%)* | Due to circumstances of pandemic and the insecurity about the situation |  | Focus on pandemic  *N=2 (8.3%)* | All attention on the pandemic |
|  | Changed residents contact  *N=4 (8.9%)* | Less contact and no affection allowed, which results in a more distant contact |  | Increased awareness on safety and hygiene measures *N=2 (8.3%)* | Being aware of them and comply to them makes the daily work more challenging |
|  | Control and instruction  *N=4 (8.9%)* | Of others (staff, relatives, residents) to comply with safety measures |  | Hysteria  *N=2 (8.3%)* | From population and the media |
|  | Residents suffer due to current situation *N=3 (6.7%)* | Because of the regulations, the social distancing, and the restrictions |  |  |  |
|  | Higher burden due to current situation *N=3 (6.7%)* | For the staff, due to the situation which is not easy to handle during work but also emotionally |  |  |  |
|  | Reduced team contact  *N=2 (4.4%)* | With staff during breaks or at lunch |  |  |  |
| **Other**  *N=17 (8.3%)* |  | Reliance on others and their behavior, priorities, safety residents, etc. | **Other**  *N=12 (11%)* |  | Leisure, personal issues, large investments, contact BAG/GD, lack of equipment, etc. |

**Table S2: What is currently particularly difficult for you in your daily work (barriers)?**

| **Nursing homes employees, n=205 (out of 296, 69.0%)** | | | **Home healthcare organization employees, n=95 (out of 131, 72.5%)** | | |
| --- | --- | --- | --- | --- | --- |
| **Themes** | **Categories** | **Descriptors** | **Themes** | **Categories** | **Descriptors** |
| **Wearing hygiene face mask**  *N=139*  *(67.8%)* | Wearing masks overall  *N=63 (45.3%)* | Without stating a reason | **Wearing hygiene face mask**  *N=62*  *(65.3%)* | Wearing masks overall  *N=23 (37.1%)* | Without stating a reason |
|  | Limited communication / mimics  *N=42 (30.3%)* | Residents with hearing problems, lip reading not possible, mimic is hidden |  | Limited communication / mimics  *N=21 (33.8%)* | Clients with hearing problems, lip reading not possible, mimic is hidden |
|  | Personal problems  *N=33 (23.7%)* | Skin irritations, lack of oxygen, glasses tarnishes, heat, fatigue |  | Personal problems  *N=14 (22.6%)* | Skin irritations, lack of oxygen, glasses tarnishes |
|  | Hindering during resident’s body care  *N=1 (0.7%)* | Lack of oxygen, feels very hot with mask |  | Hindering during client’s care  *N=4 (6.5%)* | Glasses tarnishes, feels very hot with mask |
| **Psychosocial aspects**  *N=78*  *(38.1%)* | Uncertainty  *N=18 (23.1%)* | Fear to spread Corona, to infect residents, insecurity, end of pandemic difficult to predict, handling staff absences is challenging | **Psychosocial aspects**  *N=27*  *(33.7%)* | Distance / barrier to other people  *N=10 (37.0%)* | To clients (due to safety measures, no closeness), no closeness to fellow human beings, lack of human contact |
|  | Distance / barrier to other people  *N=13 (16.7%)* | To residents (due to safety measures, no closeness possible), lack of human contact |  | Uncertainty  *N=8 (29.6%)* | Fear to spread/get corona, insecurity on pandemic course (what happens next, when is it over) |
|  | Concern for fellow human beings  *N=13 (16.7%)* | For residents; have to do without many things (visits, excursions, etc.), loneliness of residents |  | Increased effort in supporting clients  *N=4 (14.8%)* | Sensitization for safety measures is challenging, need to be psychological support for clients |
|  | Dealing with relatives / visitors  *N=13 (16.7%)* | Lack of understanding, insecurity, disregard |  | Enforcement of safety measures  *N=3 (11.1%)* | Home healthcare organization managers must motivate/convince their employees to comply with protective measures |
|  | Dealing with residents  *N=12 (15.4%)* | Explain situation, pessimism, caution |  |  |  |
|  | Enforcement / supervision  *N=9 (11.4%)* | Of workers to comply with protective measures |  |  |  |
| **Work related changes**  *N=21*  *(10.2%)* | Management / Infrastructure  *N=16 (76.2%)* | Making decisions is challenging (on measures, dealing with suspected cases), staff shortage difficult to handle, coordinating visits is challenging | **Work related changes**  *N=6*  *(6.3%)* | Management / Infrastructure  *N=4 (66.6%)* | Making decisions is challenging (assessment / implementation of events, work capability assessment), staff shortage difficult to handle |
|  | Increased workload  *N=5 (23.8%)* | Additional care for relatives during home isolation, sudden displacements of events |  | Increased workload  *N=2 (33.3%)* | More administrative work, higher complexity |
| **General safety measures (except for mask)**  *N=20*  *(9.8%)* | Keeping distance  *N=11 (55%)* | To residents is difficult | **General safety measures (except for mask)**  *N=10*  *(10.5%)* | Compliance  *N=8 (80%)* | Difficulties in keeping distance and adhere to rules |
|  | Disinfecting  *N=5 (25%)* | Skin irritations due to disinfectant |  | Other  *N=2 (20%)* | Disinfecting items correctly and in the correct order, hygiene measures overall (without explanation) |
|  | Compliance  *N=3 (15%)* | Of safety measures overall |  |  |  |
|  | Other  *N=1 (5%)* | Hygiene measures overall (without stating a reason) |  |  |  |
| **Other**  *N=26*  *(9.3%)* |  | Nothing, change of everyday life, bad protection material available, to show empathy (recognize fears of workers), lack of understanding, lack of appreciation, etc. | **Other**  *N=15*  *(15.8%)* |  | Nothing, lack of equipment, difficulties independent of corona, dissatisfaction regarding protection concepts, guidelines unclear, sudden changes occur, special situations, etc. |

**Table S3: What helps you to conduct your daily work under the current conditions (facilitators)?**

| **Nursing homes employees, n=187 (out of 297, 63.0%)** | | | **Home healthcare organization employees, n=87 (out of 131, 66.4%)** | | |
| --- | --- | --- | --- | --- | --- |
| **Themes** | **Categories** | **Descriptors** | **Themes** | **Categories** | **Descriptors** |
| **Psychosocial aspects**  *N=126*  *(67.4%)* | Team spirit  *N=36 (19.3%)* | Good working atmosphere, cohesion within the team, commitment, having fun with colleagues, supporting each other, encouragement, effective communication, professionalism, teamwork | **Psychosocial aspects**  *N=54*  *(62.1%)* | Team spirit  N=15 (17.2%) | Work atmosphere, solidarity, good mood, team, colleagues, motivate each other, cooperation |
|  | Emotional coping  *N=32 (17.1%)* | Humor, positivity, belief, self-confidence, creativity, motivate oneself, keep calm, perseverance, will |  | Emotional coping  *N=13 (14.9%)* | Positivity, humor, creativity, good mood |
|  | Hope for improvement  *N=15 (8.0%)* | Hope for vaccine/immunity, confidence due to vaccine, hope for end of pandemic, hope for improvement, daily life returns |  | Problem-oriented coping  *N=11 (12.6%)* | Rationality, acceptance of the situation, giving the best, discipline, calmness |
|  | Problem-oriented coping  *N=13 (7.0%)* | Setting priorities, acceptance of the situation, rationality, discipline, patience, calmness |  | Psychosocial support at home  *N=6 (6.9%)* | Family, friends, conversations |
|  | Conversation with colleagues  N=10 (5.3%) | Conversation with colleagues and team, interchanges |  | Sympathy from clients  N=4 (4.6%) | Appreciation and gratitude from clients |
|  | Sympathy from residents  *N=9 (4.8%)* | Sympathy from residents and visitors, contact to residents, positive feedback |  | Hope for improvement  *N=3 (3.4%)* | There is an end, hope for an end |
|  | Psychosocial support at home  *N=11 (5.9%)* | Conversations with family and friends |  | Conversations with Colleagues  N=2 (2.3%) | Conversations |
| **Work related changes**  *N=48*  *(25.7%)* | Management and infrastructure  N=21 (11.2%) | Knowledge update, protection concepts, isolation guidelines, clear rules, support from superiors | **Work related changes**  *N=27*  *(31.1%)* | Management and Infrastructure  *N=13 (14.9%)* | Organization, clear guidelines, information exchange, good protection concept |
|  | Breaks  N=15 (8.0%) | Breaks without masks, doing breaks outside, part-rime workload, rest |  | Employment in General  *N=6 (6.9%)* | Time-management, salary, home office, expertise |
|  | Passion for the profession  *N=7 (3.7%)* | Fun during work, passion for the job |  | Passion for the profession  *N=5 (5.7%)* | Passion for the job |
|  | Secure employment  *N=5 (2.7%)* | Secure workplace, salary, no short-time work, good prospects |  | Breaks  *N=3 (3.4%)* | Breaks without masks, part-time workload |
| **Safety measures**  *N= 30*  *(16.0%)* | Reduced risk of infection  N=15 (8.0%) | Measures to protect others and oneself, masks to reduce risk of infection | **Safety Measures**  *N=22*  *(25.3%)* | Hygiene measures  *N=18 (20.7%)* | Masks in general, enough protection material, hygiene measures, keeping distance, meaningful measures |
|  | Hygiene measures  N=15 (8.0%) | Keeping distance, following the hygiene rules, masks in general, enough protective material |  | Reduced risk of infection  *N=4 (4.6%)* | Self-protection with masks and hygiene measures, protection of others |
| **Others**  *N=17*  *(9.1%)* |  | Nothing, Leisure time, awareness of the danger, self-awareness | **Others**  *N=20 (23.0%)* |  | New normality, leisure time, spent time in nature, sport, habituation, nothing |

**Corona Immunitas Research Group**

The following are members of *Corona Immunitas Research Group* (including the authors of the present article), listed in alphabetical order: Emiliano Albanese, MD PhD (Institute of Public Health (IPH), Università della Svizzera Italiana (USI), Lugano, Switzerland; emiliano.albanese@usi.ch), Rebecca Amati, PhD (Institute of Public Health (IPH), Università della Svizzera Italiana (USI), Lugano, Switzerland; rebecca.amati@usi.ch), Antonio Amendola, Msc (Department of Business Economics, Health & Social Care (DEASS), University of Applied Sciences & Arts of Southern Switzerland (SUPSI), Manno, Switzerland; [antonio.amendola@supsi.ch](mailto:antonio.amendola@supsi.ch)), Daniela Anker, Msc (Population Health Laboratory (#PopHealthLab), University of Fribourg, Fribourg, Switzerland; Institute of Primary Health Care (BIHAM), University of Bern, Bern, Switzerland; [daniela.anker@unifr.ch](mailto:daniela.anker@unifr.ch)), Anna Maria Annoni, Msc (Institute of Public Health (IPH), Università della Svizzera Italiana (USI), Lugano, Switzerland; anna.maria.annoni@usi.ch), Andrew Azman, PhD (Division of Primary Care, Geneva University Hospitals, Geneva, Switzerland; Department of Epidemiology, Johns Hopkins Bloomberg School of Public Health, Baltimore, MD, USA; Institute of Global Health, University of Geneva, Geneva, Switzerland; andrew.azman@hcuge.ch), Frank Bally, MD (Institut central des hôpitaux, Hôpital du Valais, Sion, Switzerland ; frank.bally@hopitalvs.ch), Bettina Balmer, MD (Epidemiology, Biostatistics and Prevention Institute, University of Zurich, Zurich, Switzerland; balmer.schiltknecht@gmail.com), Hélène Baysson, PhD (Department of Health and Community Medicine, University of Geneva, Geneva, Switzerland; helene.baysson@unige.ch), Delphine Berthod, MD (Institut central des hôpitaux, Hôpital du Valais, Sion, Switzerland ; delphine.berthod@hopitalvs.ch), Jacob Blankenberger, BSc (Epidemiology, Biostatistics and Prevention Institute, University of Zurich, Zurich, Switzerland; jacob.blankenberger@uzh.ch), Murielle Bochud, MD PhD (Center for Primary Care and Public Health (Unisanté), University of Lausanne, Lausanne, Switzerland, murielle.bochud@unisante.ch), Patrick Bodenmann, MD Msc (Center for Primary Care and Public Health (Unisanté), University of Lausanne, Lausanne, Switzerland; patrick.bodenmann@unisante.ch), Matthias Bopp, Dr. phil.II PhD MPH (Epidemiology, Biostatistics and Prevention Institute, University of Zurich, Zurich, Switzerland; matthias.bopp@uzh.ch), Audrey Butty, MD (Center for Primary Care and Public Health (Unisanté), University of Lausanne, Lausanne, Switzerland; audrey.butty@unisante.ch), Anne Linda Camerini, PhD (Institute of Public Health (IPH), Università della Svizzera Italiana (USI), Lugano, Switzerland; anne.linda.camerini@usi.ch), Céline Cappeli, BSc (Epidemiology, Biostatistics and Prevention Institute, University of Zurich, Zurich, Switzerland; celine.capelli@uzh.ch), Cristian Carmelli, PhD (Population Health Laboratory (#PopHealthLab), University of Fribourg, Fribourg, Switzerland; cristian.carmeli@unifr.ch), Arnaud Chiolero, MD PhD, (Population Health Laboratory (#PopHealthLab), University of Fribourg, Fribourg, Switzerland; Institute of Primary Health Care (BIHAM), University of Bern, Bern, Switzerland; Department of Epidemiology, Biostatistics and Occupational Health, McGill University, Montréal, Canada; [arnaud.chiolero@unifr.ch](mailto:arnaud.chiolero@unifr.ch)), Prune Collombet, MSc (Division of Primary Care, Geneva University Hospitals, Geneva, Switzerland; [prune.collombet@hcuge.ch](mailto:prune.collombet@hcuge.ch)), Laurie Corna, PhD (Department of Business Economics, Health & Social Care (DEASS), University of Applied Sciences & Arts of Southern Switzerland (SUPSI), Manno, Switzerland; laurie.corna@supsi.ch), Jenny Crawford, MPH (Epidemiology, Biostatistics and Prevention Institute, University of Zurich, Zurich, Switzerland; jenny.crawford@uzh.ch), Luca Crivelli, PhD (Department of Business Economics, Health & Social Care (DEASS), University of Applied Sciences & Arts of Southern Switzerland (SUPSI), Manno, Switzerland; Institute of Public Health (IPH), Università della Svizzera Italiana (USI), Lugano, Switzerland; luca.crivelli@supsi.ch), Stéphane Cullati, PhD (Population Health Laboratory (#PopHealthLab), University of Fribourg, Fribourg, Switzerland; Department of Readaptation and Geriatrics, Faculty of Medicine, University of Geneva, Switzerland; [stephane.cullati@unifr.ch](mailto:stephane.cullati@unifr.ch)), Alexia Cusini, MD (Infectious Diseases Unit, Cantonal Hospital, 7000 Chur, Switzerland; [alexia.cusini@ksgr.ch](mailto:alexia.cusini@ksgr.ch)), Valérie D'Acremont, MD PhD (Center for Primary Care and Public Health (Unisanté), University of Lausanne, Lausanne, Switzerland; Swiss Tropical and Public Health Institute, University of Basel, Basel, Switzerland; valerie.dacremont@unisante.ch), Carlo De Pietro, PhD (Department of Business Economics, Health & Social Care (DEASS), University of Applied Sciences & Arts of Southern Switzerland (SUPSI), Manno, Switzerland; carlo.depietro@supsi.ch), Agathe Deschamps, MPH (Cantonal Medical Service, Neuchâtel, Switzerland; agathe.deschamps@ne.ch), Yaron Dibner (Population Epidemiology Unit, Primary Care Division, Geneva University Hospitals, Geneva, Switzerland; [yaron.diber@hcuge.ch](mailto:yaron.diber@hcuge.ch)), Sophie Droz, BA (Cantonal Medical Service, Neuchâtel, Switzerland; sophie.droz@ne.ch), Alexis Dumoulin, PhD (Institut central des hôpitaux, Hôpital du Valais, Sion, Switzerland ; alexis.dumoulin@hopitalvs.ch), Olivier Duperrex, MD Msc (Center for Primary Care and Public Health (Unisanté), University of Lausanne, Lausanne, Switzerland; [olivier.duperrex@unisante.ch](mailto:olivier.duperrex@unisante.ch)), Julien Dupraz, MD MAS (Center for Primary Care and Public Health (Unisanté), University of Lausanne, Lausanne, Switzerland; [julien.dupraz@unisante.ch](mailto:julien.dupraz@unisante.ch)), Malik Egger, MSc (Center for Primary Care and Public Health (Unisanté), University of Lausanne, Lausanne, Switzerland; malik.egger@unisante.ch), Nathalie Engler, (Cantonal Hospital St. Gallen, Clinic for Infectious Diseases and Hospital Epidemiology, St. Gallen, Switzerland; natalie.engler@kssg.ch), Adina Mihaela Epure, MD (Population Health Laboratory (#PopHealthLab), University of Fribourg, Fribourg, Switzerland; Department of Epidemiology and Health Services, Center for Primary Care and Public Health (Unisanté), University of Lausanne, Lausanne, Switzerland; adina-mihaela.epure@unifr.ch), Sandrine Estoppey, MSc (Center for Primary Care and Public Health (Unisanté), University of Lausanne, Lausanne, Switzerland; [sandrine.estoppey@unisante.ch](mailto:sandrine.estoppey@unisante.ch)), Marta Fadda, PhD (Institute of Public Health (IPH), Università della Svizzera Italiana (USI), Lugano, Switzerland; marta.fadda@usi.ch), Vincent Faivre (Center for Primary Care and Public Health (Unisanté), University of Lausanne, Lausanne, Switzerland; vincent.faivre@unisante.ch), Jan Fehr, MD (Epidemiology, Biostatistics and Prevention Institute, University of Zurich, Zurich, Switzerland; jan.fehr@usz.ch), Andrea Felappi (Center for Primary Care and Public Health (Unisanté), University of Lausanne, Lausanne, Switzerland; andrea.felappi@unisante.ch), Maddalena Fiordelli, PhD (Institute of Public Health (IPH), Università della Svizzera Italiana (USI), Lugano, Switzerland; maddalena.fiordelli@usi.ch), Antoine Flahault, MD PhD (Institute of Global Health, Faculty of Medicine, University of Geneva, Geneva, Switzerland; Division of Tropical and Humanitarian Medicine, Geneva University Hospitals, Geneva, Switzerland; Department of Health and Community Medicine, University of Geneva, Geneva, Switzerland; [antoine.flahault@unige.ch](mailto:antoine.flahault@unige.ch)), Luc Fornerod, MAS (Observatoire valaisan de la santé (OVS), Sion, Switzerland ; luc.fornerod@ovs.ch), Cristina Fragoso Corti, PhD (Department of environment construction and design (DACD), University of Applied Sciences & Arts of Southern Switzerland (SUPSI), Manno, Switzerland; cristina.fragoso@supsi.ch), Marion Frangville, Msc (Division of Primary Care, Geneva University Hospitals, Geneva, Switzerland; marion.frangville@hcuge.ch), Irène Frank, PhD (Clinical Trial Unit, Cantonal Hospital Luzern, Luzern, Switzerland; [irene.frank@luks.ch](mailto:irene.frank@luks.ch)) Giovanni Franscella, Msc (Institute of Public Health (IPH), Università della Svizzera Italiana (USI), Lugano, Switzerland; giovanni.franscella@usi.ch), Anja Frei, PhD (Epidemiology, Biostatistics and Prevention Institute, University of Zurich, Zurich, Switzerland; anja.frei@uzh.ch), Doreen Gille, MSc (Epidemiology, Biostatistics and Prevention Institute, University of Zurich, Zurich, Switzerland; doreen.gille@uzh.ch), Gisela Michel, PhD (Department of Health Sciences and Medicine, University of Luzern, Luzern, Switzerland; gisela.michel@unilu.ch)Semira Gonseth Nusslé, MSc MD (Center for Primary Care and Public Health (Unisanté), University of Lausanne, Lausanne, Switzerland; semira.gonseth-nussle@unisante.ch), Auriane Gouzowski, MA (Cantonal Medical Service, Neuchâtel, Switzerland; Auriane.gouzowski@ne.ch), Idris Guessous, MD, PhD (Division of Primary Care, Geneva University Hospitals, Geneva, Switzerland; Department of Health and Community Medicine, Faculty of Medicine, University of Geneva, Geneva, Switzerland; idris.guessous@hcuge.ch), Julien Guggisberg, BA (Cantonal Medical Service, Neuchâtel, Switzerland; Julien.guggisberg@ne.ch), Huldrych Günthard, MD (Epidemiology, Biostatistics and Prevention Institute, University of Zurich, Zurich, Switzerland; huldrych.guenthard@usz.ch), Felix Gutzwiller, MD, PhD (Epidemiology, Biostatistics and Prevention Institute, University of Zurich, Zurich, Switzerland), Medea Imboden, PhD (Swiss Tropical and Public Health Institute, Basel, Switzerland, University of Basel, Basel, Swtizerland; medea.imboden@swisstph.ch), Loussine Incici, LLM(Cantonal Medical Service, Neuchâtel, Switzerland; [loussine.incici@ne.ch](mailto:loussine.incici@ne.ch)), Emilie Jendly (Center for Primary Care and Public Health (Unisanté), University of Lausanne, Lausanne, Switzerland; emilie.jendly@unisante.ch), Ruedi Jung, Msc (Epidemiology, Biostatistics and Prevention Institute, University of Zurich, Zurich, Switzerland; ruedi.jung@uzh.ch), Christian Kahlert, MD (Cantonal Hospital St. Gallen, Clinic for Infectious Diseases and Hospital Epidemiology, St. Gallen, Switzerland; Children's Hospital of Eastern Switzerland, Infectious Diseases and Hospital Epidemiology, St. Gallen, Switzerland; christian.kahlert@kssg.ch), Laurent Kaiser, MD PhD (Geneva Center for Emerging Viral Diseases and Laboratory of Virology, Geneva University Hospitals, Geneva, Switzerland; Division of Infectious Diseases, Geneva University Hospitals, Geneva, Switzerland; Department of Medicine, Faculty of Medicine, University of Geneva, Geneva, Switzerland; laurent.kaiser@hcuge.ch), Laurent Kaufmann, MD (Cantonal Medical Service, Neuchâtel, Switzerland; laurent.kaufmann@ne.ch), Marco Kaufmann, PhD (Epidemiology, Biostatistics and Prevention Institute, University of Zurich, Zurich, Switzerland; marco.kaufmann@uzh.ch), Simone Kessler, (Cantonal Hospital St. Gallen, Clinic for Infectious Diseases and Hospital Epidemiology, St. Gallen, Switzerland; simone.kessler@kssg.ch), Philipp Kohler, MD MSc (Cantonal Hospital St. Gallen, Clinic for Infectious Diseases and Hospital Epidemiology, St. Gallen, Switzerland; philipp.kohler@kssg.ch), Susi Kriemler, MD (Epidemiology, Biostatistics and Prevention Institute, University of Zurich, Zurich, Switzerland; susi.kriemlerwiget@uzh.ch), Lauranne Lenoir, MA (Cantonal Medical Service, Neuchâtel, Switzerland; [lauranne.lenoir@ne.ch](mailto:lauranne.lenoir@ne.ch)), Sara Levati, PhD (Department of Business Economics, Health & Social Care (DEASS), University of Applied Sciences & Arts of Southern Switzerland (SUPSI), Manno, Switzerland; sara.levati@supsi.ch), Bettina Maeschli (Epidemiology, Biostatistics and Prevention Institute, University of Zurich, Zurich, Switzerland; maeschli@corona-immunitas.ch), Jean-Luc Magnin, PhD (Laboratory, HFR-Fribourg, Fribourg, Switzerland; jean-luc.magnin@h-fr.ch), Eric Masserey, MD (Cantonal Medical Office, General Health Department, Canton of Vaud, Lausanne, Switzerland; eric.masserey@vd.ch), Rosalba Morese, PhD (Institute of Public Health (IPH), Università della Svizzera Italiana (USI), Lugano, Switzerland; rosalba.morese@usi.ch), Nicolai Mösli, MD (Swiss Tropical and Public Health Institute, University of Basel, Basel, Switzerland; nicolai.moesli@swisstph.ch), Natacha Noël (Population Epidemiology Unit, Primary Care Division, Geneva University Hospitals, Geneva, Switzerland; [natacha.noel@hcuge.ch](mailto:natacha.noel@hcuge.ch)), Maëlle Orhant, BA (Cantonal Medical Service, Neuchâtel, Switzerland), Daniel Henry Paris, MD PhD (Swiss Tropical and Public Health Institute, University of Basel, Basel, Switzerland; daniel.paris@swisstph.ch), Jérôme Pasquier, PhD (Center for Primary Care and Public Health (Unisanté), University of Lausanne, Lausanne, Switzerland; jerome.pasquier@unisante.ch), Francesco Pennacchio, PhD (Division of Primary Care, Geneva University Hospitals, Geneva, Switzerland; francesco.pennacchio@hcuge.ch), Dusan Petrovic, PhD (Division of Primary Care, Geneva University Hospitals, Geneva, Switzerland; [dusan.petrovic@hcuge.ch](mailto:dusan.petrovic@hcuge.ch)), Stefan Pfister, PhD (Laboratory, HFR-Fribourg, Fribourg, Switzerland; stefan.pfister@h-fr.ch), Attilio Picazio, PhD (Population Epidemiology Unit, Primary Care Division, Geneva University Hospitals, Geneva, Switzerland), Cesarina Prandi, PhD (Department of Business Economics, Health & Social Care (DEASS), University of Applied Sciences & Arts of Southern Switzerland (SUPSI), Manno, Switzerland; cesarina.prandi@supsi.ch); Giovanni Piumatti, PhD (Institute of Public Health (IPH), Università della Svizzera Italiana (USI), Lugano, Switzerland; Giovanni.Piumatti@unige.ch), Jane Portier (Department of Primary Care, Geneva University Hospitals, Geneva, Switzerland ; jane.portier@hcuge.ch), Nicole Probst-Hensch, Dr. phil.II PhD MPH (Swiss Tropical and Public Health Institute, University of Basel, Basel, Switzerland; nicole.probst@swisstph.ch), Caroline Pugin (Population Epidemiology Unit, Primary Care Division, Geneva University Hospitals, Geneva, Switzerland; caroline.pugin@hcuge.ch), Milo Puhan, MD PhD (Epidemiology, Biostatistics and Prevention Institute, University of Zurich, Zurich, Switzerland; miloalan.puhan@uzh.ch), Thomas Radtke, PhD (Epidemiology, Biostatistics and Prevention Institute, University of Zurich, Zurich, Switzerland; thomas.radtke@uzh.ch), Aude Richard, MD MPH (Division of Primary Care, Geneva University Hospitals, Geneva, Switzerland; Institute of Global Health, Faculty of Medicine, University of Geneva, Geneva, Switzerland; aude.richard@unige.ch), Claude-François Robert, MD (Cantonal Medical Service, Neuchâtel, Switzerland; claude-francois.robert@ne.ch), Pierre-Yves Rodondi, MD (Institute of Family Medicine, University of Fribourg, Fribourg, Switzerland; pierre-yves.rodondi@unifr.ch), Nicholas Rodondi, MD (Institute of Primary Health Care (BIHAM), University of Bern, Switzerland; nicholas.rodondi@biham.unibe.ch), Eric Salberg, BA (Cantonal Medical Service, Neuchâtel, Switzerland; [Eric.salberg@ne.ch](mailto:Eric.salberg@ne.ch)), Javier Sanchis Zozaya, MD (Center for Primary Care and Public Health (Unisanté), University of Lausanne, Lausanne, Switzerland; javier.sanchis-zozaya@unisante.ch), Virginie Schlüter, MAS, MD (Center for Primary Care and Public Health (Unisanté), University of Lausanne, Lausanne, Switzerland; virginie.schlueter@unisante.ch), Valentine Schneider, MSc (Cantonal Medical Service, Neuchâtel, Switzerland; valentine.schneider@ne.ch), Amélie Steiner-Dubuis (Center for Primary Care and Public Health (Unisanté), University of Lausanne, Switzerland; amelie.steiner-dubuis@unisante.ch), Silvia Stringhini, PhD (Division of Primary Care, Geneva University Hospitals, Geneva, Switzerland; silvia.stringhini@hcuge.ch), Johannes Sumer, MD (Cantonal Hospital St. Gallen, Clinic for Infectious Diseases and Hospital Epidemiology, St. Gallen, Switzerland; johannes.sumer@kssg.ch), Ismaël Tall, MA (Cantonal Medical Service, Neuchâtel, Switzerland; [Ismael.tall@ne.ch](mailto:Ismael.tall@ne.ch)), Julien Thabard (Center for Primary Care and Public Health (Unisanté), University of Lausanne, Lausanne, Switzerland; julien.thabard@unisante.ch), Mauro Tonolla, PhD (Department of environment construction and design (DACD), University of Applied Sciences & Arts of Southern Switzerland (SUPSI), Manno, Switzerland; mauro.tonolla@supsi.ch), Nicolas Troillet, MD Msc (Institut central des hôpitaux, Hôpital du Valais, Sion, Switzerland ; nicolas.troillet@hopitalvs.ch), Agne Ulyte, MD (Epidemiology, Biostatistics and Prevention Institute, University of Zurich, Zurich, Switzerland; agne.ulyte@uzh.ch), Sophie Vassaux, MSc (Center for Primary Care and Public Health (Unisanté), University of Lausanne, Lausanne, Switzerland; sophie.vassaux@unisante.ch), Thomas Vermes, Msc (Swiss Tropical and Public Health Institute, University of Basel, Basel, Switzerland; thomas.vermes@swisstph.ch), Fabian Vollrath, Msc, (Epidemiology, Biostatistics and Prevention Institute, University of Zurich, Zurich, Switzerland; vollrath@corona-immunitas.ch),Viktor von Wyl, PhD (Epidemiology, Biostatistics and Prevention Institute, University of Zurich, Zurich, Switzerland; viktor.vonwyl@uzh.ch), Erin West, Msc (Epidemiology, Biostatistics and Prevention Institute, University of Zurich, Zurich, Switzerland; erinashley.west@uzh.ch), Ania Wisniak, MD (Division of Primary Care, Geneva University Hospitals, Geneva, Switzerland; Institute of Global Health, Faculty of Medicine, University of Geneva, Geneva, Switzerland; anna.wisniak@unige.ch), Maria-Eugenia Zaballa, PhD (Population Epidemiology Unit, Primary Care Division, Geneva University Hospitals, Geneva, Switzerland; mariaeugenia.zaballa@hcuge.ch), Claire Zuppinger, MSc (Center for Primary Care and Public Health (Unisanté), University of Lausanne, Lausanne, Switzerland; claire.zuppinger@unisante.ch)
